# Maternal dietary methionine restriction alters the expression of energy metabolism genes in the duckling liver

**DOI:** 10.1186/s12864-022-08634-1

**Published:** 2022-05-30

**Authors:** Aurélie Sécula, Hervé Chapuis, Anne Collin, Lisa E. Bluy, Agnès Bonnet, Loys Bodin, Laure Gress, Alexis Cornuez, Xavier Martin, Cécile M. D. Bonnefont, Mireille Morisson

**Affiliations:** 1 GenPhySE, Université de Toulouse, INRAE, ENVT, F-31326 Castanet Tolosan, France; 2 Present Address: IHAP, Université de Toulouse, INRAE, ENVT, Toulouse, France; 3 INRAE, Université de Tours, BOA, 37380 Nouzilly, France; 4 UEPFG INRAE Bordeaux-Aquitaine (Unité Expérimentale Palmipèdes à Foie Gras), Domaine d’Artiguères 1076, route de Haut Mauco, F-40280 Benquet, France

**Keywords:** Duck, Methyl donor, Nutritional programming, Differentially expressed genes

## Abstract

**Background:**

In mammals, the nutritional status experienced during embryonic development shapes key metabolic pathways and influences the health and phenotype of the future individual, a phenomenon known as nutritional programming. In farmed birds as well, the quantity and quality of feed offered to the dam can impact the phenotype of the offspring. We have previously reported that a 38% reduction in the intake of the methyl donor methionine in the diet of 30 female ducks during the growing and laying periods - from 10 to 51 weeks of age - reduced the body weight of their 180 mule ducklings compared to that of 190 ducklings from 30 control females. The maternal dietary methionine restriction also altered the hepatic energy metabolism studied in 30 of their ducklings. Thus, their plasma glucose and triglyceride concentrations were higher while their plasma free fatty acid level was lower than those measured in the plasma of 30 ducklings from the control group. The objective of this new study was to better understand how maternal dietary methionine restriction affected the livers of their newly hatched male and female ducklings by investigating the hepatic expression levels of 100 genes primarily targeting energy metabolism, amino acid transport, oxidative stress, apoptotic activity and susceptibility to liver injury.

**Results:**

Sixteen of the genes studied were differentially expressed between the ducklings from the two groups. Maternal dietary methionine restriction affected the mRNA levels of genes involved in different pathways related to energy metabolism such as glycolysis, lipogenesis or electron transport. Moreover, the mRNA levels of the nuclear receptors PPARGC1B, PPARG and RXRA were also affected.

**Conclusions:**

Our results show that the 38% reduction in methionine intake in the diet of female ducks during the growing and egg-laying periods impacted the liver transcriptome of their offspring, which may explain the previously observed differences in their liver energy metabolism. These changes in mRNA levels, together with the observed phenotypic data, suggest an early modulation in the establishment of metabolic pathways.

**Supplementary Information:**

The online version contains supplementary material available at 10.1186/s12864-022-08634-1.

## Background

The effects of maternal nutrition on offspring phenotypes have been largely documented and reviewed in recent years in human and rodents [1–4] as well as in farmed animals [5–7] including poultry [8, 9]. In particular, the nutritional status experienced early in life, i.e. during embryonic and fetal development, interacts with major metabolic pathways and shapes the health and phenotypes of the individual into adulthood, a phenomenon known as nutritional programming.

In birds, *in ovo* manipulation of nutrients by either injection of a nutrient or removal of a part of a component is a direct way to impact the offspring phenotypes [10, 11]. For example, Willems and coauthors removed a part of the albumen to explore the lasting impacts of protein under-nutrition in layer-type hens. They reported a lower hatching weight and hepatic proteome changes in chicks hatched from albumen deprived eggs [12] and differential gene expression in the hepatic transcriptome of the adult hens from albumen-deprived eggs [13]. These adult hens laid smaller eggs and had a lower laying rate and a higher number of second grade eggs, a consequence of early protein under-nutrition [14]. Altogether, this group demonstrated long-lasting effects of nutritional programming induced by early protein under-nutrition on production performances in layer-type hens. It has also been reported that early methyl donor availability plays critical roles in hepatic carbohydrate and lipid metabolism. For example, *in ovo* injection of betaine was shown to affect hepatic cholesterol metabolism in newly hatched chicks [15] and to protect from corticosterone-induced hepatic steatosis [16].

However, the quantity and quality of the feed offered to female birds can also have an impact on the performances of the offspring. For example, when betaine was added to the diet of hens, it altered the expression of genes in the liver of their chicks [17]. In a previous study, we investigated the effects of a reduced level of dietary methionine (Met) on laying performances of female common ducks *Anas platyrhynchos* and its impacts on the phenotypes of their newly hatched mule ducklings [18]. Indeed, the male mule duck is the male sterile inter-generic hybrid offspring of a female common duck and a Muscovy drake (*Cairina moschata*). It benefits from heterosis effects that increase the production of fatty liver (hepatic steatosis) induced by overfeeding and is therefore widely used for foie gras production in France [19]. In our previous study [18], the restricted group of females received Met-restricted diets (R group) containing 0.25% of Met whereas the control group received control diets (C group) containing 0.40% of Met that meets Met requirements, during the growing and laying periods, from 10 to 51 weeks of age. Thus, females in the R group laid eggs of lower weight and containing less albumen, thus inducing a lower availability of nutrients for embryonic development. The ducklings that were the offspring of the females from the R and C groups were subsequently assigned to R and C groups, respectively. The male and female newly hatched ducklings in the R group showed a reduced body weight when compared to those of the C group and a tendency to an increased ratio of liver weight to body weight. Moreover, their plasma alanine transaminase (ALT) activity was reduced and their plasma alkaline phosphatase (ALP) activity showed a tendency to be reduced too. Their plasma concentrations of glucose and triglycerides (TG) were higher whereas their plasma level of free fatty acids (FFA) decreased. These observations suggested altered hepatic energy metabolism in male and female newly hatched ducklings from the Met-restricted dams. Based on literature data, this alteration could be a consequence of a nutritional programming following the reduced availability of Met -that is a methyl donor- in the maternal diet and a lower availability of nutrients during embryonic development. In this context, the objective of the present study was to further explore the effects of the maternal dietary Met restriction by comparing the level of expression of 100 genes in the liver from male and female newly hatched ducklings either offspring of the Met-Restricted or Control dams. These target genes were mainly related to energy metabolism, amino acid transport, oxidative stress, apoptotic activity and susceptibility to liver injury.

## Results

The objective of this study was to compare the hepatic transcript levels of 100 target genes in the livers of C group and R group ducklings to provide information on how maternal methionine restriction affected hepatic energy metabolism in male and female ducklings from the Met-restricted dams.

The normalized relative expression of the 100 target genes was analyzed in 38 duckling livers from both groups (R group versus C group) after a qqnorm transformation. However, 13 of the 100 genes studied and 2 of the 38 liver cDNA samples showed more that 25% of missing data and were removed from the study (see Method section). Moreover, another cDNA sample was also removed from the data set because it showed data points that differed significantly from other observations. The results are thus given for the 87 remaining genes and the 35 remaining liver cDNA samples which are from 9 male and 8 female ducklings from the C group and 10 male and 8 female ducklings from the R group.

### Liver samples were classified into four subgroups according to the maternal diet and to the sex of the ducklings

**Fig. 1:**
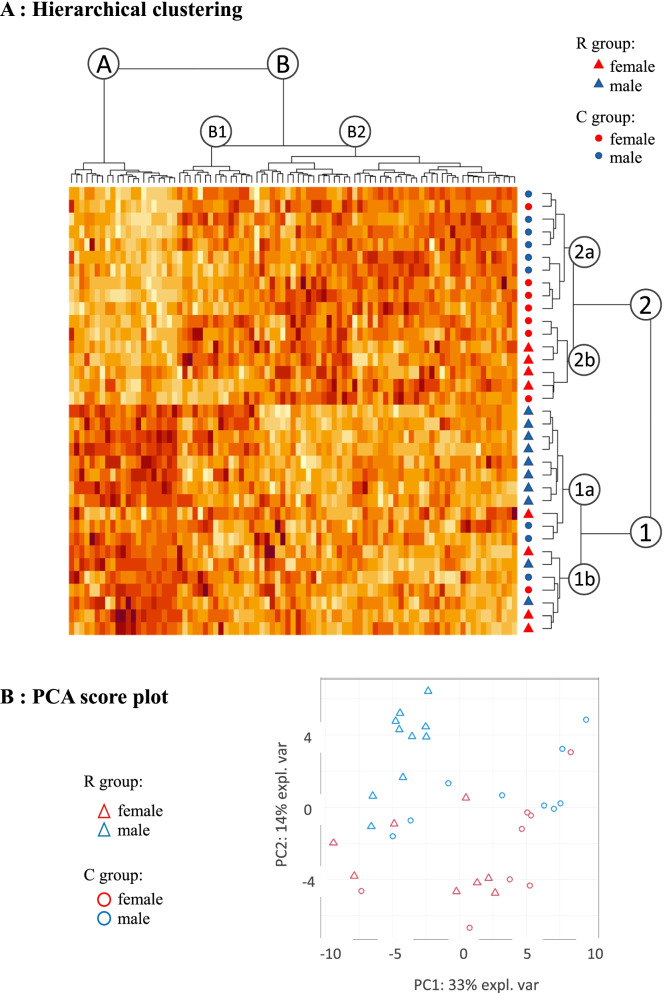
Exploratory data analyses of the 87 genes. The ducklings from R group and C group are represented with triangles and circles, respectively. The females are in red and the males in blue. **A**. Hierarchical Cluster Analysis of the gene expressions. In the heatmap, the duckling liver samples and the genes are arranged in rows and columns, respectively. The yellow, orange and red colors correspond to the low, median and high values of the qqnorm transformed normalized relative expressions of the studied genes. The clusters corresponding to duckling liver samples are named 1, 1a, 1b, 2, 2a and 2b. The clusters corresponding to genes are named A, B, B1 and B2. **B**. Score plot of the PCA along the 2 first principal components. The two first principal components summarized respectively 33% (horizontal axis) and 14% (vertical axis) of the whole variability

First, a hierarchical clustering was performed to define groups of genes and animals with similar expression patterns. The ducklings were roughly separated according to the two maternal diets within two clusters numbered 1 and 2 on rows in Fig. 1A. The cluster 1 was mainly composed of ducklings belonging to the R group whereas the cluster 2 was mainly composed of ducklings belonging to the C group. In each case, only 4 ducklings were not grouped to their initial group. The cluster 1 was divided into 2 sub-clusters called 1a and 1b. The sub-cluster 1a contained 11 samples corresponding to 8 male ducklings from the R group, 2 male ducklings from the C group and 1 female duckling from the R group. The sub-cluster 1b contained 7 samples that were 3 female and 2 male ducklings from the R group and 1 male and 1 female ducklings from the C group. The cluster 2 was divided into 2 sub-clusters called 2a and 2b. The sub-cluster 2a contained 10 samples corresponding to 6 male and 4 female samples from the C group. The sub-cluster 2b contained 7 female samples, 4 from the R group and 3 from the C group.

Based on their relative expression level, the 87 genes were grouped into 2 main clusters on the columns, named A and B. The cluster A was composed of 21 genes overexpressed in samples of cluster 1, mainly composed of ducklings belonging to the R group. The cluster B was divided into 2 sub-clusters called B1 and B2. The sub-cluster B1 was composed of 15 genes whose expression levels did not differ between the previously defined duckling clusters 1 and 2. In contrast, the large sub-cluster B2 showed 51 genes that attempted to be overexpressed in samples from cluster 2, which mainly consisted of ducklings belonging to the C group.

The score plot (distribution of individuals) of the PCA (Principal Component Analysis) along the 2 first principal components (horizontal and vertical axes) is given on Fig. 1B. The samples were first separated by the maternal diet on the horizontal axis and then by the sex of the ducklings on the vertical axis. The two first principal component summarized respectively 33% (horizontal axis) and 14% (vertical axis) of the whole variability.

Altogether, the samples were separated not only according to the maternal diet but also according to the sex of the ducklings thus defining four subgroups i.e. males from the R group (MR), females from the R group (FR), males from the C group (MC) and females from the C group (FC).

### Maternal dietary Met restriction altered expression of energy metabolism genes

Of the 87 genes studied, the normalized and transformed relative expressions were used to investigate the differences in gene expression between the liver samples of the offspring of the two diet groups (C group versus R group) (Additional Table 1). Data were analyzed with a linear mixed model that included the maternal diet, the sex of the duckling, and the interaction between them as fixed effects, as well as the duckling -associated to its relationship matrix- as a random effect. The Table 1 describes the 27 genes showing a significant difference (corrected *P*-value < 0.05) imputable either to the maternal diet (Diet *P*-value (BH); 16 significant genes) or/and to the sex of the duckling (Sex *P*-value (BH); 15 significant genes). Four of them (*GPAM, PGM1, ELOVL6, and PRKAA1*) showed significant differences for both the maternal diet and the sex of the ducklings. Finally, no gene had a significant interaction between duckling sex and maternal diet effects. Seven genes that tend to be differentially expressed (*P*-value (BH) between 0.05 and 0.10) between maternal diets (2 genes) or between duckling sexes (6 genes) are added in Table 1 and identified by a star. For each gene, least square means and standard deviations are given for the two groups of maternal diet (R group and C group) and for the two duckling sexes. Hereafter, the focus will be on the 16 differentially expressed genes (DEGs) for the maternal diet effect. Nine of them were down-regulated (*NDUFA4, COX2, ENO1, MTTP, PRKAA1, RXRA, CYTB, NUDFB6* and *PPARGC1B*) whereas seven were upregulated (*GPAM*, *PGM1*, *ELOVL6*, *PGK1*, *UGDH*, *BCL2A1* and *PPARG*) in the R group samples when compared the C group samples.

**Table 1:** Differentially expressed genes (DEGs) in the liver of ducklings

GENE	R group	C group	Females	Males	Diet	Sex	Sex*Diet
	LsMeans ± SD	LsMeans ± SD	LsMeans ± SD	LsMeans ± SD	***P***-value (BH)	***P***-value (BH)	***P***-value (BH)
***GPAM***	0.39 ± 0.16	− 0.52 ± 0.17	− 0.59 ± 0.17	0.47 ± 0.15	< 0.01	**< 0.01**	0.28
*NDUFA4*	− 0.61 ± 0.19	0.64 ± 0.19	− 0.02 ± 0.2	0.05 ± 0.18	< 0.01	0.98	0.99
***PGM1***	0.53 ± 0.15	− 0.65 ± 0.15	− 0.53 ± 0.16	0.41 ± 0.14	< 0.01	**< 0.01**	0.78
*COX2*	− 0.55 ± 0.24	0.77 ± 0.28	0.01 ± 0.26	0.2 ± 0.25	0.01	0.93	0.85
***ELOVL6***	0.51 ± 0.27	− 0.5 ± 0.31	− 0.46 ± 0.26	0.47 ± 0.25	0.01	**< 0.01**	0.92
*ENO1*	−0.54 ± 0.24	0.51 ± 0.27	−0.1 ± 0.25	0.07 ± 0.23	0.01	0.81	0.85
*MTTP*	−0.48 ± 0.21	0.55 ± 0.22	0.1 ± 0.23	−0.02 ± 0.2	0.01	0.87	1.00
*PGK1*	0.49 ± 0.25	−0.46 ± 0.27	−0.29 ± 0.25	0.32 ± 0.23	0.01	0.07 *	0.29
***PRKAA1***	−0.49 ± 0.2	0.4 ± 0.2	−0.48 ± 0.21	0.39 ± 0.19	0.01	**0.02**	0.83
*RXRA*	−0.78 ± 0.3	0.51 ± 0.24	−0.77 ± 0.31	0.49 ± 0.23	0.01	0.11	1.00
*UGDH*	0.48 ± 0.21	−0.53 ± 0.22	−0.2 ± 0.22	0.15 ± 0.2	0.01	0.42	0.83
*CYTB*	−0.6 ± 0.28	0.52 ± 0.33	−0.15 ± 0.29	0.07 ± 0.27	0.02	0.54	0.70
*BCL2A1*	0.34 ± 0.21	−0.43 ± 0.21	−0.33 ± 0.22	0.25 ± 0.2	0.03	0.13	0.70
*NDUFB6*	−0.4 ± 0.22	0.42 ± 0.22	−0.08 ± 0.23	0.1 ± 0.21	0.03	0.81	0.70
*PPARG*	0.47 ± 0.22	−0.5 ± 0.24	−0.1 ± 0.23	0.07 ± 0.22	0.03	0.81	0.87
*PPARGC1B*	−0.43 ± 0.23	0.43 ± 0.23	0 ± 0.23	0 ± 0.22	0.03	0.98	0.87
*BMF*	−0.48 ± 0.3	0.46 ± 0.34	−0.27 ± 0.3	0.25 ± 0.29	0.06 *	0.18	0.78
*PPARA*	−0.35 ± 0.22	0.33 ± 0.22	−0.29 ± 0.23	0.27 ± 0.21	0.10 *	0.23	0.79
***ELAVL1***	0.2 ± 0.24	−0.35 ± 0.28	−0.67 ± 0.24	0.53 ± 0.24	0.22	**< 0.01**	0.99
***HMGCR***	0.1 ± 0.33	− 0.25 ± 0.37	−0.67 ± 0.32	0.51 ± 0.31	0.47	**< 0.01**	0.12
***MEF2C***	0 ± 0.19	− 0.12 ± 0.19	−0.71 ± 0.2	0.59 ± 0.18	0.75	**< 0.01**	1.00
***SCD1***	0.32 ± 0.26	− 0.44 ± 0.31	−0.52 ± 0.26	0.4 ± 0.26	0.10	**< 0.01**	0.70
***TALDO1***	0.31 ± 0.25	− 0.37 ± 0.29	−0.58 ± 0.25	0.51 ± 0.24	0.10	**< 0.01**	0.70
***VLDLR***	−0.23 ± 0.3	0.01 ± 0.34	0.58 ± 0.29	−0.8 ± 0.29	0.66	**< 0.01**	0.99
***PCK1***	−0.28 ± 0.31	0.1 ± 0.35	0.36 ± 0.31	−0.54 ± 0.3	0.47	**0.01**	0.85
***FASN***	0.28 ± 0.27	−0.38 ± 0.31	−0.43 ± 0.27	0.33 ± 0.26	0.14	**0.02**	0.08
***MAPK1***	0.19 ± 0.3	−0.38 ± 0.35	−0.57 ± 0.31	0.38 ± 0.29	0.32	**0.02**	0.85
***ABCA1***	0.05 ± 0.29	−0.27 ± 0.32	−0.54 ± 0.29	0.32 ± 0.28	0.55	**0.04**	0.84
***DGAT2***	0.06 ± 0.24	−0.12 ± 0.24	−0.49 ± 0.24	0.43 ± 0.23	0.71	**0.04**	0.85
*ALDOB*	−0.34 ± 0.25	0.36 ± 0.28	0.36 ± 0.26	−0.35 ± 0.24	0.13	0.06 *	0.85
*SDHA*	−0.24 ± 0.22	0.16 ± 0.24	−0.45 ± 0.24	0.37 ± 0.22	0.35	0.06 *	0.85
*DHCR24*	0.11 ± 0.2	−0.15 ± 0.2	−0.37 ± 0.21	0.33 ± 0.2	0.44	0.07 *	0.08
*HADH*	−0.32 ± 0.23	0.21 ± 0.23	−0.44 ± 0.25	0.33 ± 0.21	0.30	0.07 *	0.83
*LDHA*	−0.13 ± 0.37	0.01 ± 0.43	0.26 ± 0.37	−0.38 ± 0.35	0.86	0.07 *	0.28

Genes are listed according to whether they are differentially expressed for maternal diet (first part of the table) or for duckling sex (second part of the table and/or in bold). The corrected *P*-value with Benjamini-Hochberg (BH) procedure of the diet effect, the sex effect and their interaction are presented

The star (*) indicates genes with a *P*-value (BH) between 0.05 and 0.1. For each gene, least square means (Ls-Means) and standard deviations (SD) are presented for the two maternal diet groups (R group and C group) and for both sexes

**Fig. 2:**
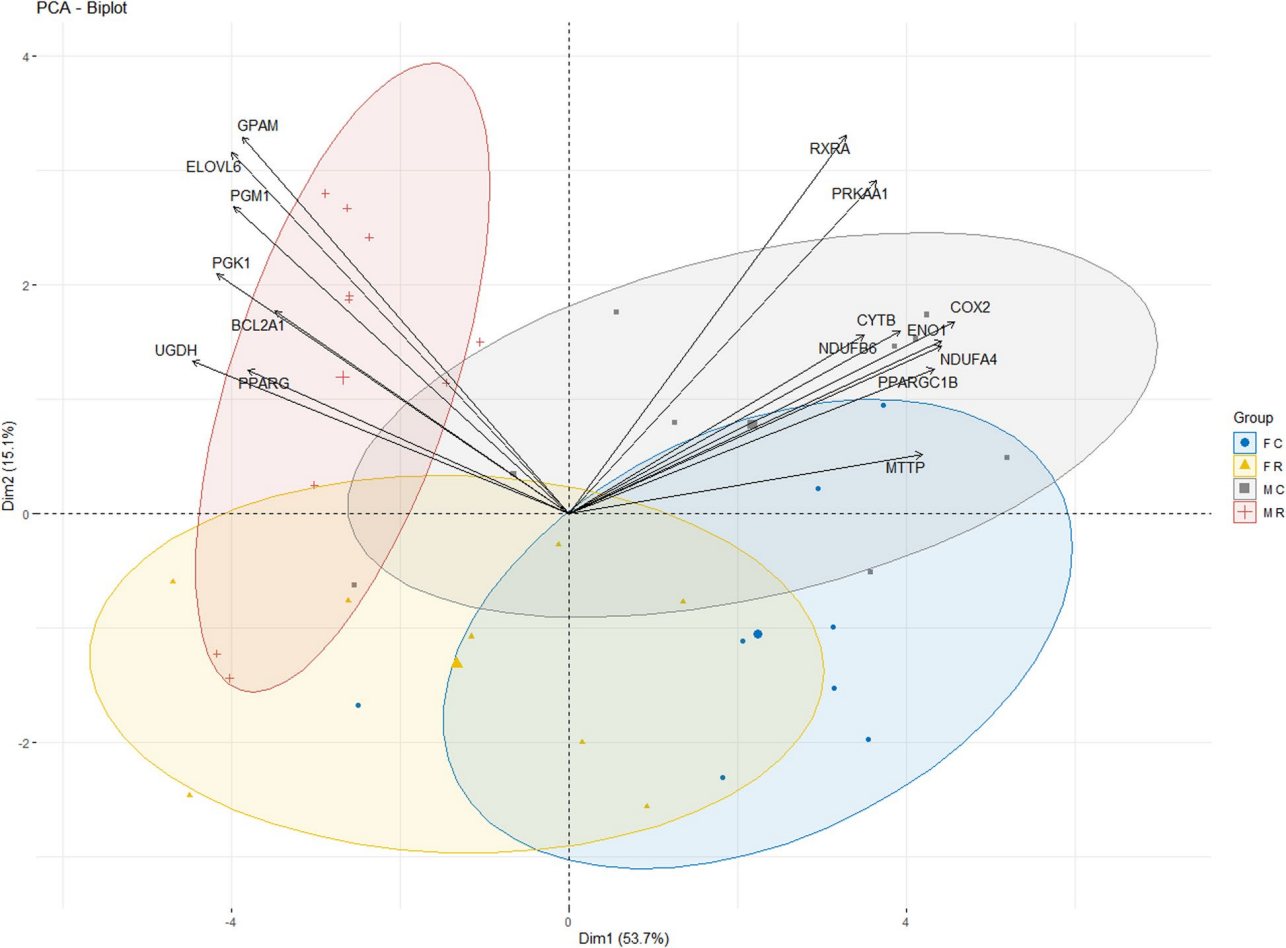
Biplot of principal component analysis. PCA was performed on the data of the 16 DEGs for the diet effect. The male ducklings from the R group (MR) and the C group (MC) are represented in red crosses and grey squares, respectively and the females from the R group (FR) and the C group (FC) are in yellow triangles and blue circles, respectively. The ellipses to gather the groups were added. The two first principal components explain 53.7 and 15.1% of the whole variability, respectively. The correlation circle showed correlations between the DEGs and the two main principal components and displayed an opposite regulation pattern between the 9 down-regulated genes (on the right side of the figure) and the 7 up-regulated ones (on the left side of the figure), when compared the R group to the C group samples

The Fig. 2 shows the results of the Principal Component Analysis (PCA) performed on the 16 DEGs for the maternal diet effect. The first principal component (horizontal axis) explained 53.7% of the whole variability and discriminated the samples according to the diet received by the female ducks (R group versus C group). The second principal component (vertical axis) explained 15.1% of the whole variability and discriminated the samples according to the sex of the ducklings. This is in concordance with the fact that 4 out of the 16 DEGs showed a significant effect of the sex of the ducklings and a fifth one tended to be differentially expressed according to the sex of the ducklings (Table 1). Again, the expression variability divided the samples into the four same sub-groups: MR, FR, MC and FC. In addition, the PCA bi-plot showed correlations between the DEGs and the two main principal components and confirmed the opposite regulation pattern between the 9 down-regulated genes and the 7 up-regulated ones reported when compared R group and C group samples in Table 1.

### Correlations between the DEGs and the duckling phenotypic traits revealed differences in liver metabolism

Phenotypic traits were measured in a previous study on the same newly hatched mule ducklings [18] and the main results are summarized in Table 2. These traits included body weight, liver weight, percentages of liver lipids and liver dry mater (DM), plasma activities of ALP, ALT and AST as well as plasma concentrations of glucose, TG and FFA.

**Table 2:** Effects of maternal dietary Met restriction on duckling traits (from Bodin et al., 2019 [18])

	Treatment						
		R group	C group				
	n	Mean ± SD	n	Mean ± SD	P_Diet_	P_sex_	P_interaction_
Body weight (g)	180	33.0 ± 0.9	190	35.2 ± 0.9	< 0.001	NS	NS
Liver weight (g)	28	1.51 ± 0.08	21	1.40 ± 0.11	NS	0.001	NS
Liver: BW (%)	28	4.30 ± 0.17	21	3.92 ± 0.20	0.07	0.06	NS
Liver lipids (%)	28	17.23 ± 1.43	19	17.67 ± 1.44	NS	NS	NS
Liver dry matter (%)	28	41.10 ± 0.73	20	41.21 ± 1.10	NS	NS	NS
Plasma Glucose (mMol/L)	23	16.39 ± 1.88	26	10.63 ± 2.38	0.03	NS	NS
Plasma FFA (mMol/L)	28	0.27 ± 0.05	27	0.55 ± 0.05	0.01	0.07	NS
Log Plasma TG	27	0.55 ± 0.19	27	- 0.09 ± 0.21	0.01	0.01	NS
Log Plasma ALP	28	5.36 ± 0.09	24	5.62 ± 0.10	0.07	< 0.001	NS
Log Plasma ALT	28	2.90 ± 0.09	23	3.32 ± 0.09	0.002	0.01	NS
Log Plasma AST	27	4.42 ± 0.19	27	4.69 ± 0.21	NS	0.006	NS

Numbers, means, and standard errors of the measured traits as well as the significance of the differences between means are given. *P*-values < 0.05 were considered significant

**Fig. 3:**
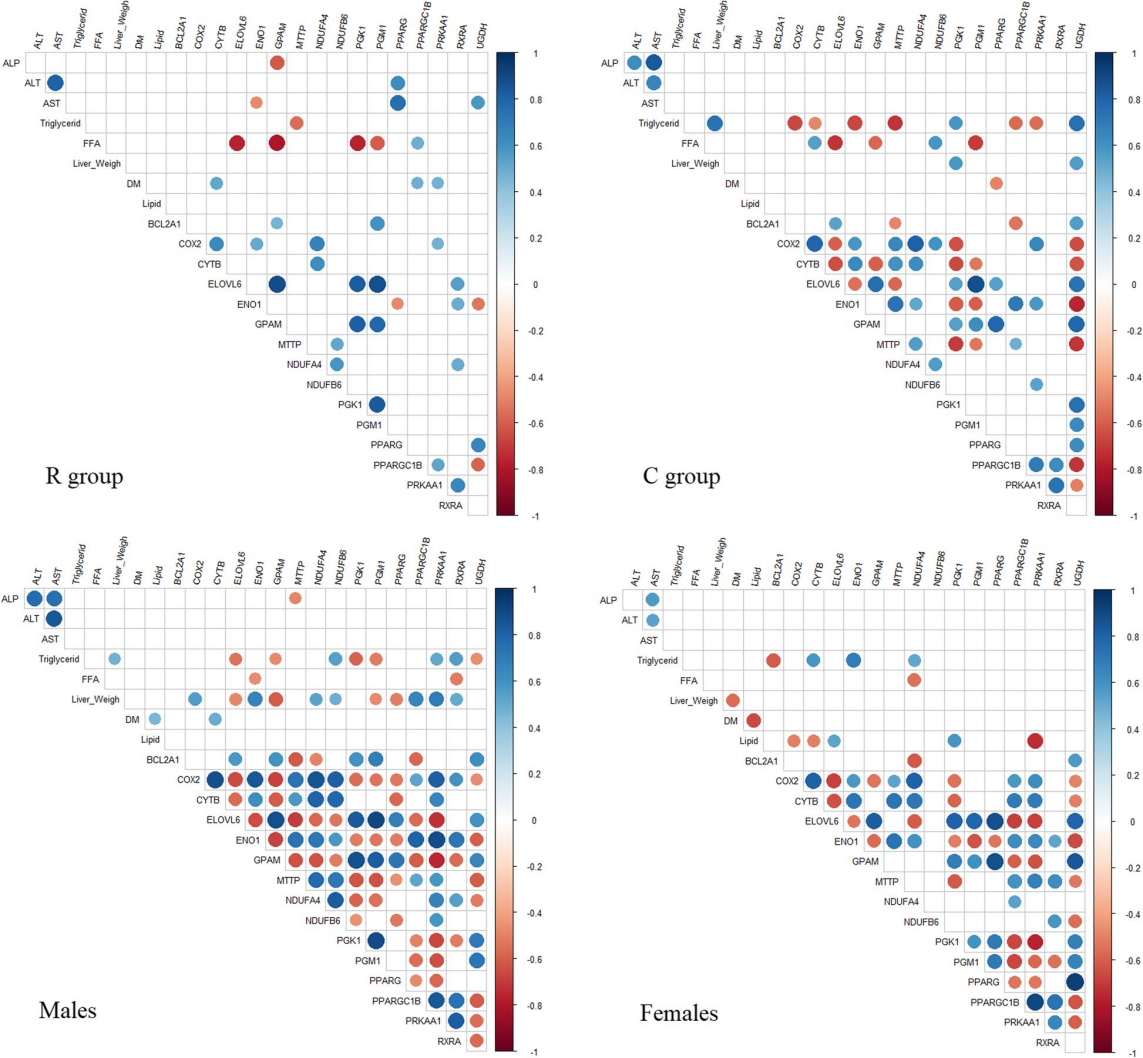
Correlation matrices of the transcript level of the 16 DEGs between diets and the phenotypic traits of the ducklings. The correlation matrices were plotted for the R group (*n* = 18), the C group (*n* = 17), and the males (*n* = 19) and the female ducklings (*n* = 16). Phenotypic traits are liver weight, percentages of liver lipids and liver dry mater (DM), plasma activities of ALP, ALT and AST, plasma cholesterol, glucose, triglyceride and free fatty acid (FFA) concentrations. The values used for the 16 DEGs were the imputed normalized expression and the values for the phenotypic data were the raw values. The color scale indicates the strength of the correlation; blue for a positive correlation and red for a negative one. Only the significant correlations (with a *P*-value < 0.05) were plotted

The Fig. 3 shows the correlation matrices of the hepatic mRNA levels of the 16 DEGs between the two maternal diets and the phenotypic traits of the ducklings in the R and C groups and then in males and females.

The correlation matrices differed strongly between the ducklings from the R group and the ones from the C group. Briefly, the number of significant correlations is lower in the R group than in the C group. In particular, the plasma concentration of TG is only correlated to *MTTP* in the R group (− 0.55) whereas it is correlated to 8 genes in the C group (6 negative correlations: -0.66 *COX2*, − 0.49 *CYTB*, − 0.66 *ENO1*, − 0.71 *MTTP*, − 0.56 *PPARGC1B* and − 0.55 *PRKAA1* and 2 positive correlations: 0.58 *PGK1* and 0.75 *UGDH*). On the contrary the plasma activities of ALP, ALT and AST were correlated to 0 DEG in the C group and to 1 DEG for ALP (− 0.61 *GPAM*), to 1 DEG for ALT (0.63 *PPARG*) and to 3 DEGs for AST (− 0.47 *ENO1*, 0.75 *PPARG* and 0.58 *UGDH*) in the R group. The correlations of the plasmatic content of FFA are quite similar between the R group and the C group with a negative correlation with *ELOVL6* (− 077, and − 0.72, in the R and C group respectively), *GPAM* (− 0.80 and − 0.58, in the R and C group respectively) and *PGM1* (− 0.60 and − 0.70, in the R and C group respectively). But two significant correlations were detected only in the R group (− 0.76 *PGK1* and 0.49 *PPARGC1B*) and two other ones only in the C group (0.55 *CYTB* and 0.58 *NDUFB6*). The correlations between the hepatic content and the 16 hepatic DEGs were low. The hepatic lipid content was correlated to 0 DEG in both groups, the liver weight was only correlated to 2 genes in the C group (0.57 *PGK1* and 0.55 *UGDH*) and the DM content was correlated to 3 DEGs in the R group (0.51 *CYTB*, 0.48 *PPARGC1B* and 0.47 *PRKAA1*) and to 1 DEG in the C group (− 0.50 *PPARG*).

In addition, the number of correlations between DEGs is higher in the C group than in the R group. For example, *UGDH* is correlated to 12 and 3 DEGs, *COX2* to 9 and 4, *CYTB* to 9 and 2, *ELOVL6* to 10 and 4, *ENO1* to 10 and 4, in the C group and R group, respectively.

Furthermore, the comparison of males and females reveals that the phenotypes are more correlated to the DEGs in males than in females. Actually, the plasmatic content of TG is correlated to 8 DEGs in the males (5 negative correlations: -0.53 *ELOVL6*, − 0.48 *GPAM*, − 0.58 *PGK1*, − 0.52 *PGM1*, − 0.46 *UGDH* and 3 positive correlations: 0.54 *NDUFB6*, 0.51 *PRKAA1* and 0.56 *RXRA*) and 4 DEGs in the females (− 0.61 *BCL2A1*, 0.59 *CYTB*, 0.68 *ENO1* and 0.52 *NDUFA4*) and the liver weight is correlated to 11 DEGs in the males (7 positive correlations: 0.55 *COX2*, 0.67 *ENO1*, 0.54 *NDUFA4*, 0.48 *NDUFB6*, 0.66 *PPARGC1B*, 0.70 *PRKAA1*, 0.51 *RXRA* and 4 negative correlations: -0.49 *ELOVL6*, − 0.61 *GPAM*, − 0.47 *PGM1* and − 0.50 *PPARG*) and 0 DEG in females. On the contrary the hepatic lipid content is correlated to 0 DEG in males and to 5 DEGs in females (− 0.50 *COX2*, − 0.51 *CYTB*, − 0.73 *PRKAA1*, 0.53 *ELOVL6* and 0.59 *PGK1*). In addition, the correlation between DEGs is strong in both males and females except for *BCL2A1* and *NDUFB6*, where the correlations with the other DEGs are stronger in males than in females.

The same correlation matrices were obtained in the four subgroups (MR, MC, FR and FC) and are presented in Additional Figure 1. Briefly, it can be noticed that the correlations between DEGs are stronger in the males from the C group (MC) compared to those in the males from the R group (MR). Moreover, the correlation matrices differ not only between groups (MR versus MC and FR versus FC) but also between sexes (MR versus FR and MC versus FC), which is consistent with the results found in the exploratory data analyses (Fig. 1) and the Principal Component Analysis (Fig. 2).

## Discussion

The liver is the main tissue for lipid synthesis in birds. In adult mule ducks, the capacity to accumulate and store lipids -mainly triglycerides- in liver, is enhanced by overfeeding that leads to hepatic steatosis for fatty liver production. In the newly hatched mule ducklings, prior to the first feeding, body weight was about 6% lower while the liver to body weight ratio tended to be about 10% higher in ducklings from R group compared to those of the C group. Moreover, plasmatic parameters suggested altered hepatic energy metabolism in male and female ducklings from the Met-restricted dams (Table 2).

**Fig. 4:**
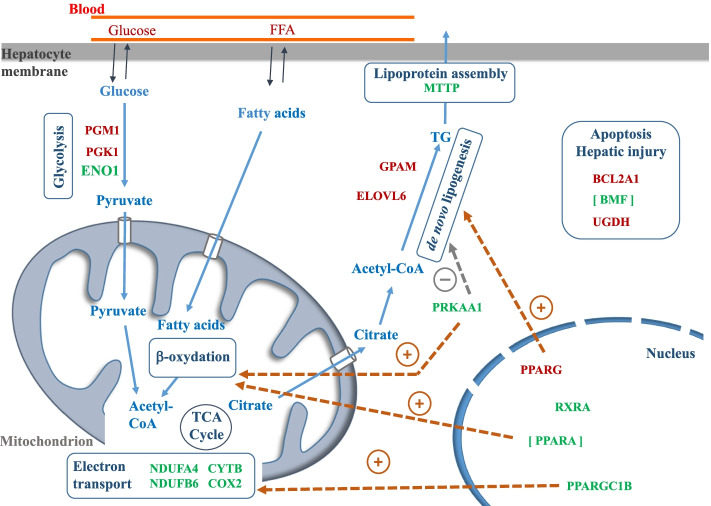
Schematic representation of the role of DEGs assigned to energetic metabolism and their regulation in newly hatched ducklings from the R group. The main metabolic pathways impacted by the maternal methionine deficiency are noted (glycolysis, electron transport, de novo lipogenesis, etc.). Up-regulated and down-regulated DEGs in ducklings issued from dams receiving Met-restricted diet are in red and green, respectively. The genes [*BMF*] and [*PPARA*] tended to be significant for the diet effect and were added to the Figure but kept in brackets. PPARA promotes fatty acid oxidation whereas PPARG favors de novo lipogenesis (dashes of brown color). PRKAA1 drives fatty acid oxidation (dashes of brown color) and decreases de novo lipogenesis (dashes of grey color). PPARGC1B regulates mitochondrial energy transfers (dashes of brown color)

We therefore studied the livers of newly hatched ducklings, questioning the extent to which nutritional programming may have altered the expression level of hepatic genes in ducklings from dams fed reduced Met diets. Indeed, such a change in gene expression, if accompanied by a change in the synthesis of associated proteins, could alter metabolic pathways and lead to long-term effects on the ability of overfed animals to develop hepatic steatosis. The Fig. 4 gives a schematic representation of the role of the 16 DEGs and the 2 genes which tended to be differentially expressed (*PPARA* and *BMF, P*-value (BH) ≤ 0.10) in the liver of newly hatched R group ducklings. The up-regulated and down-regulated DEGs being respectively in red and green.

Although the dry matter content and the lipid content of the livers of R group ducklings were similar to the ones of C group ducklings, the maternal dietary methionine deficiency affected the mRNA level of genes involved in different pathways linked to energy metabolism such as glycolysis, lipogenesis, and mitochondrial electron transport. Thus, among the three DEGs involved in glycolysis, one was down-regulated (*ENO1*) and two were up-regulated (*PGM1* and *PGK1*) in the R group. Moreover, *COX2* (*MT-CO2*), *NDUFA4*, *NDUFB6* and *CYTB* (*MT-CYB*), involved in the electron transport were all down-regulated in the R group ducklings suggesting a less active mitochondrial electron transport chain in this group. Furthermore, *ELOVL6* and *GPAM* were up-regulated suggesting a higher amount of TG in livers of the R group ducklings. In this group, the high content of glucose in plasma may increase the glycolysis activity and the pyruvate production. Then, in the mitochondria, the pyruvate may be converted into Acetyl-CoA that may in turn be converted in citrate by the tricarboxylic acid cycle (TCA cycle). Citrate may then be exported from the mitochondria and used as precursor of the de novo lipogenesis. The expression level of *MTTP* whose protein controls lipoprotein assembly was down-regulated in the R group which may have led to limited lipid export from the liver. Moreover, *PRKAA1* encodes for the catalytic subunit of the 5′-prime-AMP-activated protein kinase (AMPK). In hepatocytes, PRKAA1 drives fatty acid oxidation and decreases lipogenesis, protecting against fatty liver disease [20]. In the current study, reduced *PRKAA1* mRNA levels in R group ducklings may also have contributed to higher lipogenesis. However, none of these genes showed a correlation between their expression level and the measured percentage of liver lipids.

The *PPARGC1B* gene (PPARG Coactivator 1 Beta, also known as *PGC1B*) encodes a nuclear protein which belongs to the peroxisome proliferator-activated receptor-g coactivators (PGC-1 s). They coactivate transcription factors and nuclear receptors, including PPARG, to control expression of genes involved, among other things, in electron transport, fatty acid oxidation or de novo lipogenesis. Indeed, PPARG is a master regulator of lipogenesis which has been described to promote lipid storage in the liver [21–23]. PPARA is also a member of the nuclear receptor PPAR family and is highly expressed in the liver where it is the main regulator of fatty acid catabolism by regulating the transcription of genes involved in β-oxidation. Both PPARA and PPARG are activated upon binding by fatty acids ligands. In the absence of ligand, they associate to RXR partner and bind to corepressor complex and repress target genes. RXRA (Retinoid X Receptor Alpha) is a member of the RXR family and the RXRA/PPARA heterodimer is required for PPARA transcriptional activity on fatty acid oxidation genes [24]. The coactivator PPARGC1B regulates mitochondrial energy transfers in the liver by positively regulating the expression of electron transport genes such as COX2, and mitochondrial β-oxidation [25]. In the current study, *PPARGC1B* (*PGC1B*) and *RXRA* were down-regulated and *PPARA* tended to be down-regulated too whereas *PPARG* was up-regulated in the liver of ducklings from the R group. Thus, in general in R group ducklings, the down-regulation of *RXRA* and *PPARGC1B –* and possibly also the one of *PPARA* -might have limited energy dissipation through fatty acid oxidation and thermogenesis, while the up-regulation of *PPARG* might have promoted energy storage through increased lipogenesis. *RXRA* showed no significant correlation with the traits measured in the ducklings of both groups. On the contrary *PPARG* was positively correlated with plasma activity of AST and ALT in the R group and negatively correlated to the liver DM content in the C group. *PPARGC1B* was positively correlated with plasma FFA and liver DM content in the R group and negatively correlated with plasma TG in the C group (Fig. 3).

Apart from these results concerning the regulation of energy metabolism in the R group, other regulatory pathways were interestingly modulated by the maternal methionine supply in the offspring liver, especially concerning apoptosis. Indeed, both BMF and BCL2A1 belong to the BCL-2 protein family which governs the mitochondria-dependent pathway for apoptosis. Many Bcl-2 family proteins are localized to the membranes of mitochondria and both pro- and anti-apoptotic Bcl-2 family proteins exist. *BMF* encodes a protein that has been shown to bind Bcl-2 proteins and functions as pro-apoptotic protein whereas BCL2A1 reduces the release of pro-apoptotic cytochrome c from mitochondria and block caspase activation and thus functions as an anti-apoptotic protein. In our study, *BMF* tended to be down-regulated whereas *BCL2A1* was up-regulated suggesting a metabolism turn towards a less apoptotic activity in ducklings from the R group. Moreover, *UGDH* was up-regulated in the R group samples, suggesting a better detoxification activity in this group. Indeed, *UGDH* encodes UDP-glucose dehydrogenase that converts UDP-glucose (UDP-Glc) to UDPglucuronic acid (UDP-GlcA). Interestingly UDP-GlcA is needed for detoxification of toxic compounds in liver via glucuronidation [26]. Overall, these results suggest a regulation of apoptotic pathways and tissue damage by the maternal diet. This is consistent with a lower activity of ALT activity and a tendency to a reduced activity of ALP activity in the plasma of ducklings from the R group suggesting a fetal programming towards a reduced susceptibility to hepatic injury.

As evocated in the Background section, the availability of methyl donors in the maternal diet and its effects on liver metabolism during embryonic development are well studied, especially in mammals where reviews highlight the interactions between one-carbon metabolism, epigenetic mechanisms and energy metabolism [27–29]. In birds, Ruquian Zhao’s team has studied the effects of betaine supplementation, either by *in ovo* injection or by addition in the maternal diet. This team showed that *in ovo* injection of betaine affects hepatic cholesterol metabolism in newly hatched chicks [15] and protects them from corticosterone-induced hepatic steatosis [16]. Moreover, the authors also reported that the decrease of hepatic cholesterol content in the offspring of hens supplemented with betaine was accompanied with epigenetic modulation of *SREBP2* and *CYP7A1* genes [30] and that a change in the hepatic expression of the *Dio1*, *BHMT* and *DNMT1* genes was observed in female chicks from betaine-supplemented hens [17]. Additionally, the maternal supplementation led to hepatoprotective effects when their male chicks were exposed chronically to corticosterone [31].

Our results clearly show that in ducks, the availability of methyl donors in the maternal diet also impacts liver metabolism and hepatoprotective effects. Based on what has been described in mammals, we hypothesize that the reduced availability of methyl donors in the maternal diet may have altered the activity of one-carbon metabolism in the duckling liver. This would have affected the availability of methyl groups and impacted epigenetic mechanisms (DNA and histone methylation) in the liver of the R group ducklings, leading to altered methylation of gene promotors and inducing changes in energy metabolism pathways. In order to investigate this hypothesis, we are currently studying the impact of the maternal dietary restriction on the transcripts of genes involved in one-carbon metabolism and some epigenetic mechanisms.

Interestingly, throughout this study, the results also highlighted the extent to which the hepatic metabolism of mule ducklings is sex-dependent. A substantial proportion of the genes are differentially expressed according to the sex in many tissues and sexual dimorphism of gene expression in the liver has already been reported [32] including sex differences in PPAR signaling pathways in rodent models [33]. Thus, the application of a nutritional deficiency on metabolisms that already differ, leads to different impacts and sex differences in response to developmental programming as already reviewed by Aiken and Ozanne [34] and more recently by McCabe and coauthors [35]. In our study, some of the measured traits showed a sex effect [18] (Table 2) and, moreover, a number of the studied genes were differentially expressed depending of the sex of the ducklings (Table 1). Therefore, the liver metabolism of female ducklings differed from that of male ducklings in both groups of ducklings (R and C groups). Indeed, the correlation matrices of the two sexes differed within each group, i.e. MR versus FR and MC versus FC (Additional Fig. 1).

## Conclusions

In conclusion, in the present study, we compared the hepatic expression levels of 100 target genes in ducklings from female ducks fed either a control diet or a methionine-restricted diet to look for transcriptomic evidence of early metabolic programming in R ducklings. We also sought to correlate differences in gene expression with phenotypic data already reported in a previous article [18]. The current study has shown that the transcriptome of the offspring is influenced, and therefore modifiable, by variations in the maternal diet, such as a 38% reduction in methionine content. Thus, the differential expression level of the 16 DEGs, if linked to a differential production level of the associated proteins, may contribute to changes in energy metabolism and a reduced apoptotic activity in the livers of group R ducklings. These early changes in mRNA levels together with the observed phenotypic data, suggest modulations in the establishment of early metabolic pathways. This nutritional programming experiment was obtained on mule ducklings that benefit from heterosis from the Muscovy drake and the female common duck for the interest of mule ducks in fatty liver production. However, it would be of main interest to study the nutritional programming experiment on the pure common duck to know if the liver metabolism is impacted similarly in pure breed.

## Methods

### Animals and experimental design

Experimental procedures and animal care were conducted in compliance with the European Communities Council Directive 2010/63/EU. The experiment was conducted at the Ducks and Goose Experimental Facility – INRAE, UEPFG, (Benquet, France) that received the accreditation number B40–037-1. The protocol and procedures were approved by the French Minister of Higher Education, Research and Innovation (authorization APAFIS#1847-2015092213418825v2).

The experimental design has already been described [18]. Briefly, 60 female common ducks received adequate level of Met until the age of 10 weeks and were then divided into two groups. They were fed experimental diets from 10 to 51 weeks of age. Met is the first main limiting amino acid in a typical corn−soybean diet for poultry [36]. In laying ducks, the recommendations vary from 0.40 to 0.45% [37–40]. Thus, two levels of total Met were used for the experimental diets: 0.25% for Met-restricted diets (R Group) and 0.40% for control diets (C Group) that meets Met requirements for female laying ducks (Additional Table 2). The mule duckling production was performed by 2 artificial inseminations per week between 34 and 36 weeks of age, with the semen of 15 Muscovy drakes not subjected to any dietary treatment and fed commercial diets. The eggs were incubated for 28 days at 37.6 °C and 60% average relative humidity throughout the entire incubation period (incubator Sologne, La Nationale, Briaire, France). They were then put in a hatcher (hatcher Bretagne, La Nationale, Briaire, France) for 4 days at 37.3 °C and 80% average relative humidity. Ducklings traits were recorded at hatching. The ducklings that were the offspring of the females from the R and C groups are subsequently assigned to R and C groups, respectively.

Phenotypic traits of ducklings were recorded at hatching on 180 and 190 ducklings from the R and C groups respectively, as reported in Bodin et al., 2019 [18]. Moreover, a total of 58 ducklings were sacrificed by cervical dislocation at hatching (12 females and 16 males from the C group and 15 females and 15 males from the R group). These ducklings did not receive any feed before being sacrificed. Their liver weight was recorded. In some cases, the gallbladder has been damaged and for this reason, only the livers from 8 females and 13 males from the C group and 15 females and 15 males from the R group, that were satisfactorily retrieved were immediately immersed in liquid nitrogen before being transferred to a − 80 °C freezer.

### RNA extraction and reverse transcription

Frozen liver samples from newly hatched ducklings of both sexes and of both control and restricted maternal diet groups (10 males and 8 females in the C group and 10 males and 10 females in the R group) were ground using a Retsch grinder at 30 Hz for 45 seconds, in liquid nitrogen. Next, 80 to 100 mg of tissue powder were processed as previously described [41] for RNA extraction and purification using the TRIzol® method (Invitrogen, California, USA) followed by a column from Nucleospin RNA kit (Macherey Nagel, France) and following the manufacturer’s instructions. The on-column DNAse treatment was done with 20 μl of rDNAse (Macherey Nagel) and 80 μl of reaction buffer for 20 min to avoid DNA contamination as recommended [42, 43]. The total RNA was quantified using a NanoDrop 8000 spectrophotometer (Thermo Fisher, Illkirch, France) and stored at − 80 °C. Its integrity was controlled by electrophoresis and using an Agilent 2100 Bioanalyzer, with the RNA 6000 Nano Lab Chip Kit (Agilent Technologies, Massy, France). Reverse transcription was carried out immediately after the quality control evaluation, and the same amount of total RNA was used for all experimental samples, in accordance with recommendations [44]. The reaction used the RNase H-MMLV reverse transcriptase SuperScriptTM II (Invitrogen, California, USA), RNasin® Ribonuclease inhibitor (Promega Corporation, USA) and oligo (dT)15 (Sigma Aldrich, France). The cDNAs were then diluted in RNase free water and stored at − 80 °C.

### Primer design and qPCR validation

The study targeted 100 genes selected from the literature for being related to energy metabolism as well as to amino acid transport, oxidative stress, apoptotic activity and susceptibility to liver damage. Sequences were obtained either from *Anas platyrhynchos* when available, or from *Gallus gallus* on NCBI [45] and/or Ensembl [46] databases. The two primers used for each gene (Additional Table 3) were designed in exons, but each on either side of an intron, and for an annealing temperature of 60 °C, using either Primer3Plus [47] or LightCycler® Probe Design Software 2.0 (Roche Applied Science). The primer sequences were blasted to the databases to confirm that they were specific to the gene in question. PCR products were first subjected to 2% agarose gel electrophoresis to confirm amplicon size. Next, the primer pairs showing a specific band and no primer dimers were selected to be tested by qPCR, using SYBR green fluorescence detection (Applied Biosystems) and a QuantStudio6 (Thermo Fisher Scientific). Each primer pair was tested on four serial dilutions of a pool of cDNA (cDNA of all the animals used in the study) to obtain a standard curve and check the PCR efficiency, each point being done in duplicate. The conditions were: 50 °C for 2 min, denaturation at 95 °C for 10 min, 40 cycles of 15 s at 95 °C and 1 min at 60 °C. A gradual temperature increasing from 60 °C to 95 °C was added to analyze the melting curves and detect primer dimers. The Cq values and the PCR efficiency were obtained directly from the QuantStudio Real-Time PCR software v1.3.

### Identification of potential reference genes

The expressions of 9 potential reference genes were tested for their stability in livers of newly hatched mule ducklings. Primer sequences were either from Chapman et al., 2016 [48] (designed in *Anas platyrhynchos*: *ALB, GAPDH, NDUFA10* and *RPS13*), from Staines et al., 2016 [49] (designed in *Gallus gallus*: *HMBS* and *TBP*) or newly designed in *Anas platyrhynchos* (*HPRT1, POLA1* and *TUBA1C*). These 9 primer pairs were tested by qPCR on liver cDNA from 8 ducklings of both sexes and of both maternal diet groups (C group and R group) with SYBR green fluorescence detection, on a QuantStudio6. The selection of the 5 most stable genes (*GAPDH, HMBS, NDUFA10, RPS13, TBP*), highlighted in dark grey in Additional Table 3) was done with the package SLqPCR on RStudio [50].

### Quantitative PCR and gene expression analysis

The quantification of gene expression was performed using the 96.96 Dynamic Array integrated fluidic circuits (IFCs) and the BioMark HD system from Fluidigm as previously described [51].

In this paper we present the study of transcripts of genes involved in energy metabolism in the liver of newly hatched ducklings but the full experiment was conducted on 168 liver samples (38 samples of newly hatched duckling livers and 130 samples of older duck livers) and a total of 170 genes, either targeting energy metabolism for this study (100 genes) or playing a role in one-carbon metabolism (70 genes), as well as five reference genes identified in the previous step. As the technology used did not allow all samples and genes to be analyzed at the same time, care was taken to randomize the samples onto two arrays and the genes into each specific target amplification (STA). Thus, a total of four chips were made. In this study, we focus our interest on the 38 liver samples from newly hatched ducklings and 100 genes involved in energy metabolism.

For each chip, the first step consisted of a STA with 14 cycles performed on the cDNA samples, a calibrator sample (a pool of the 38 cDNA samples), a cDNA pool of the 168 cDNA samples in five-fold serial dilutions (to determine the PCR amplification efficiency), a duck genomic DNA control, an internal control (human genomic DNA) and a negative control (TE). The first STA was performed with a pre-amplification primer mix that contained primers for the five potential reference genes and 85 target genes (among those 53 are some of this study). A second STA contained primers for the five potential reference genes and other 85 target genes (among those 47 are some of this study). The resulting STA cDNA samples were treated according to Bonnet et al., 2013 [51].

Data were analyzed with the Fluidigm Digital PCR Analysis software (version 4) using the linear (derivative) baseline correction method and the auto (global) cycle threshold (Cq) method. The data were pre-processed before expression analysis. Indeed, the cycle threshold values (Ct) recorded from amplifications whose melting curves showed either abnormal Tm (melting temperature) or double peaks (corresponding to a mixture of expected and aberrant PCR products) or a high baseline, were removed. To measure the efficiency (E) of PCR for each gene, the slope of the standard curve obtained with serial dilutions of the 168 cDNA pool was used and genes with less than three dilution points were eliminated. Finally, all genes with an efficiency greater than 2.2 or less than 1.7 were removed from the analysis.

The relative expression (RE_(i,j)_) for each gene (i) and each sample (j) was calculated as proposed by Pfaffl [52]: RE_(i,j)_ = Eff_(i)_^^(Cq(i,cal) – Cq(i,j))^ where Cq_(i,cal)_ is the Cq for the gene (i) determined for the calibrator sample (a pool of the 38 cDNA samples). To determine the reference genes, the stability of the five potential genes was tested using the GeNorm (version 3.4) algorithm [50]. The three most stable genes were chosen as references genes (*GAPDH, RPS13* and *TBP*). Finally, the relative expression (RE_(i,j)_) for each gene (i) and each sample (j) was normalized with the geometric average expression of the three most stable reference genes as proposed by Vandesompele et al. [50].

In the end, 13 of the 100 genes studied and 2 of the 38 liver cDNA samples showed more that 25% of missing data and were removed from the study. Moreover, another cDNA sample was also removed from the data set because it showed data points that differed significantly from other observations. The 13 removed genes are highlighted in light grey in Additional Table 3. The 35 remaining liver cDNA samples are from 9 male and 8 female ducklings from the C group and 10 male and 8 female ducklings from the R group.

### Statistical analyses

For the 87 remaining genes, the few missing values were imputed inside each group with similar sex and maternal diet using the function imputPCA with 3 principal components of the missMDA package of the R software [53]. These normalized and imputed relative expressions were then transformed using the function qqnorm(Y)$x to make the data follow a centered reduced normal distribution. These transformed data were then used to describe the whole dataset.

First a heatmap was obtained by using the R gplots package thus defining groups of animals and genes with similar expression patterns and a Principal Component Analysis (PCA) was performed with the package MixOmics of R software [54] on the 87 genes. The individuals were plotted on the two first principal components of the PCA score plot.

Then, ANOVAs were conducted on the transformed normalized relative expressions of the 87 genes using a linear mixed model fitted with the ASReml software [55] that included the maternal diet, the sex of the duckling, and the interaction between them as fixed effects, as well as the duckling associated to its relationship matrix as a random effect. Genes with a significant difference - diet *P*-value < 0.05 assessed after a Benjamini-Hochberg (1995) [56] correction, in order to account for multiple tests and named (diet *P*-Value (BH)) - were selected and considered as differentially expressed genes (DEGs). The effect of sex on gene expression was also evaluated (sex *P*-value (BH)), as well as the interaction between sex and diet effects (sex*diet *P*-value (BH)). For each gene, least square means and standard deviations were calculated for the 2 groups of maternal diet (R group and C group), for the 2 sexes, and finally for the four subgroups of interest, i.e., males in the R group and C group and females in the R group and C group (Additional Table 1).

Further analyses were performed on the 16 DEGs for the diet effect. Another PCA was performed on the 16 DEGs with the package MixOmics of R software [54], using the qqnorm transformed normalized relative expressions. The biplot of variables and samples from the two first principal components was obtained with the package Factoextra of R. Concentration ellipses were plotted around each group mean points with a confidence level of 0.75 using the package ellipse.

Then, correlation matrices were performed using phenotypic traits of the ducklings and the 16 DEGs for the diet effect, using the imputed normalized relative expressions to be consistent with the phenotypic data which were also not transformed.

The correlation matrices were plotted with the package Hmisc of R using the functions rcorr and corrplots [57, 58] and only the correlations with a *P*-value < 0.05 were reported.

Lastly, this study was conducted and is reported in accordance with the ARRIVE guidelines [59].

## Supplementary Information

**Additional file 1: Table 1.** Differentially expressed genes (DEGs) in the liver of ducklings.

**Additional file 2: Table 2.** Composition of the Met-restricted diets of the dam common ducks (from Bodin et al., 2019 [18]).

**Additional file 3: Table 3.** Description of the 100 targeted genes and the 5 potential reference genes.

**Additional file 4: Figure 1.** Correlation matrices of the transcript level of the 16 DEGs between diets and the phenotypic traits of the ducklings.

## Data Availability

The datasets supporting the conclusions of this article are included within the article and its three Additional Tables and one Additional Figure.

## Abbreviations

ALB: Albumin
ALDOB: Aldolase, Fructose-Bisphosphate B
ALP: Alkaline Phosphatase
ALT: Alanine Transaminase
ANOVA: Analysis of variance
AST: Aspartate aminotransferase
BCL2A1: BCL2 related protein A1
BMF: Bcl2 Modifying Factor
cDNA: Complementary deoxyribonucleic acid
COX2: Cytochrome c Oxidase subunit II
Cq: Cycle threshold
Ct: Cycle threshold value
CYTB: Cytochrome b
DEG: Differentially Expressed Gene
DGAT2: Diacylglycerol O-acyltransferase 2
DHCR24: 24-Dehydrocholesterol Reductase
DM: Dry mater
DNAse: Deoxyribonuclease
ELAVL1: ELAV Like RNA Binding Protein 1
ELOVL6: ELOVL Fatty Acid Elongase 6
ENO1: Enolase 1
FASN: Fatty acid synthase
FC: Females from the C group
FFA: Free Fatty acids
FR: Females from the R group
GAPDH: Glyceraldehyde-3-phosphate dehydrogenase
GPAM: Glycerol-3-phosphate acyltransferase, mitochondrial
HMBS: Hydroxymethylbilane synthase
HMGCR: 3-hydroxy-3-methylglutaryl-CoA reductase
HPRT1: Hypoxanthine Phosphoribosyltransferase 1
LDHA: Lactate dehydrogenase A
MC: Males from the C group
Met: Methionine
MR: Males from the R group
mRNA: Messenger ribonucleic acid
MTTP: Microsomal triglyceride transfer protein
NDUFA10: NADH:Ubiquinone Oxidoreductase Subunit A10
NDUFA4: NDUFA4, mitochondrial complex associated
NDUFB6: NADH:Ubiquinone Oxidoreductase Subunit B6
PCA: Principal Component Analysis
PCK1: Phosphoenolpyruvate carboxykinase 1
PCR: Polymerase Chain Reaction
PGC-1: Peroxisome proliferator-activated receptor-g coactivator
PGK1: Phosphoglycerate kinase 1
PGM1: Phosphoglucomutase 1
POLA1: DNA Polymerase Alpha 1, Catalytic Subunit
PPARA: Peroxisome proliferator activated receptor alpha
PPARG: Peroxisome proliferator activated receptor gamma
PPARGC1B: PPARG Coactivator 1 Beta
PRKAA1: Protein Kinase AMP-Activated Catalytic Subunit Alpha 1
qPCR: Quantitative Real-Time PCR
RE: Relative expression
RNAse: Ribonuclease
RPS13: Ribosomal Protein S13
RXRA: Retinoid X Receptor Alpha
SCD1: Stearoyl-CoA desaturase (delta-9-desaturase)
STA: Specific target amplification
TALDO1: Transaldolase 1
TBP: TATA box binding protein
TCA cycle: Tricarboxylic acid cycle
TE: Tris-EDTA buffer
TG: Triglycerides
Tm: Melting temperature
TUBA1C: Tubulin Alpha 1c
UGDH: UDP-glucose 6-dehydrogenase
VLDLR: Very low density lipoprotein receptor
