# Supplementary material for: Maternal dietary methionine restriction alters the expression of energy metabolism genes in the duckling liver
Source: BMC Genomics. 2022 May 30;23:407. doi: 10.1186/s12864-022-08634-1 (PMC9150296; doi:10.1186/s12864-022-08634-1)
Supplement: Supplementary file 1 — Additional file 1: Table 1. Differentially expressed genes (DEGs) in the liver of ducklings. [file 12864_2022_8634_MOESM1_ESM.docx]

Genes are listed according to whether they are differentially expressed for maternal diet (first part of the table), for duckling sex (second part of the table and/or in bold) or not (third part of the table). The corrected P-values with Benjamini-Hochberg (BH) procedure of the diet effect, the sex effect and their interaction are presented. The star (*) indicates genes with a P-value (BH) between 0.05 and 0.1. For each gene, least square means (Ls-Means) and standard deviations (SD) are presented for the two maternal diet groups (R group and C group), for both sexes and for the four subgroups of interest, i.e. females in R group and C group and males in R group and C group.
