# Supplementary material for: Maternal dietary methionine restriction alters the expression of energy metabolism genes in the duckling liver
Source: BMC Genomics. 2022 May 30;23:407. doi: 10.1186/s12864-022-08634-1 (PMC9150296; doi:10.1186/s12864-022-08634-1)
Supplement: Supplementary file 2 — Additional file 2: Table 2. Composition of the Met-restricted diets of the dam common ducks (from Bodin et al., 2019 [18]). [file 12864_2022_8634_MOESM2_ESM.docx]

|  |  |  |  |  |  |  |
| --- | --- | --- | --- | --- | --- | --- |
|  |  | Growing diet |  | Reproduction diet |  |  |
|  |  | (10-15 weeks) |  | (beyond 16 weeks) |  |  |
|  |  |  |  |  |  |  |
|  |  |  |  |  |  |  |
|  |  | INGREDIENTS (g/kg) | | |  |  |
|  |  |  |  |  |  |  |
|  | Maize | 501 |  | 461.8 |  |  |
|  | Soybean meal | 196.3 |  | 222.3 |  |  |
|  | Wheat | 200 |  | 200 |  |  |
|  | Wheat bran | 53.3 |  | 26.8 |  |  |
|  | Soybean oil | 20 |  | 10 |  |  |
|  | Phosphate Dicalcium | 13.4 |  | 16.8 |  |  |
|  | Limestone | 7.78 |  | 54.8 |  |  |
|  | Oligo vitamins premix^1^ | 5 |  | 4 |  |  |
|  | NaCl | 3 |  | 3 |  |  |
|  | Lys Hcl | 0.22 |  | 0.5 |  |  |
|  |  |  |  |  |  |  |
|  |  |  |  |  |  |  |
|  |  | EXPECTED NUTRITIONAL COMPOSITION | | |  |  |
|  |  |  |  |  |  |  |
|  | Metabolisable Energy (Kcal/kg) | 2,914 |  | 2,715 |  |  |
|  | Protein (g/kg) | 160 |  | 164.5 |  |  |
|  | Lys (g/kg) | 7.7 |  | 8.63 |  |  |
|  | Met (g/kg) | 2.55 |  | 2.60 |  |  |
|  | Met + Cysteine (g/kg) | 5.57 |  | 5.63 |  |  |
|  | Try (g/kg) | 1.8 |  | 1.87 |  |  |
|  | Thr (g/kg) | 5.93 |  | 6.12 |  |  |
|  | Ca (g/kg) | 8.28 |  | 26.5 |  |  |
|  | P (g/kg) | 3.33 |  | 3.79 |  |  |
|  | Choline chloride (g/kg) | 0.55 |  | 0. 55 |  |  |
|  |  |  |  |  |  |  |
|  |  |  |  |  |  |  |

^1^Oligo-vitamins premix for reproduction diet (0.40%):10,000 IU vitamin A (retinol); 4,000 UI vitamin D3 (cholecalciferol); 80 mg vitamin E (tocopherol); 4 mg vitamin K3 (menadione); 4 mg vitamin B1 (thiamine); 6 mg vitamin B2 (riboflavin); 80 mg vitamin B3 (PP, niacin); 20 mg vitamin B5 (Ca panthotenate); 6 mg vitamin B6 (pyridoxine); 0.2 mg vitamin B8 (biotin, H); 2 mg vitamin B9 (folic acid); 0.02 mg vitamin B12 (cyanocobalamin); 440 mg choline; 40 mg Fe (FeCO3); 16 mg Cu (CuSO4); 64 mg Mn (MnO); 72 mg Zn (ZnSO4); 0.4 mg Co (CoCO3); 2 mg I (Ca(IO3)2); 0.2 mg Se (Na2SeO3); 2.29 g Ca (CaCO3).

Oligo-vitamins premix (0.50%) for growing diet (0.50%): 20% of the previous premix was added to the growing diet to reach to 0.50%.

Pure DL Met was added to the Met-restricted diets to reach a concentration of 4.2 g/kg in the growing and reproduction control diets (C).

The Met contents of these experimental diets were measured (INVIVO LABS SAS; Chateau Thierry, France). The results were in accordance with the expected levels (0.24%, 0.25%, 0.40%, and 0.40% for the R growing, R reproduction, C growing, and C reproduction diets, respectively, after correction by the dry matter of the feeds).
