# Supplementary material for: Maternal dietary methionine restriction alters the expression of energy metabolism genes in the duckling liver
Source: BMC Genomics. 2022 May 30;23:407. doi: 10.1186/s12864-022-08634-1 (PMC9150296; doi:10.1186/s12864-022-08634-1)
Supplement: Supplementary file 3 — Additional file 3: Table 3. Description of the 100 targeted genes and the 5 potential reference genes. [file 12864_2022_8634_MOESM3_ESM.docx]

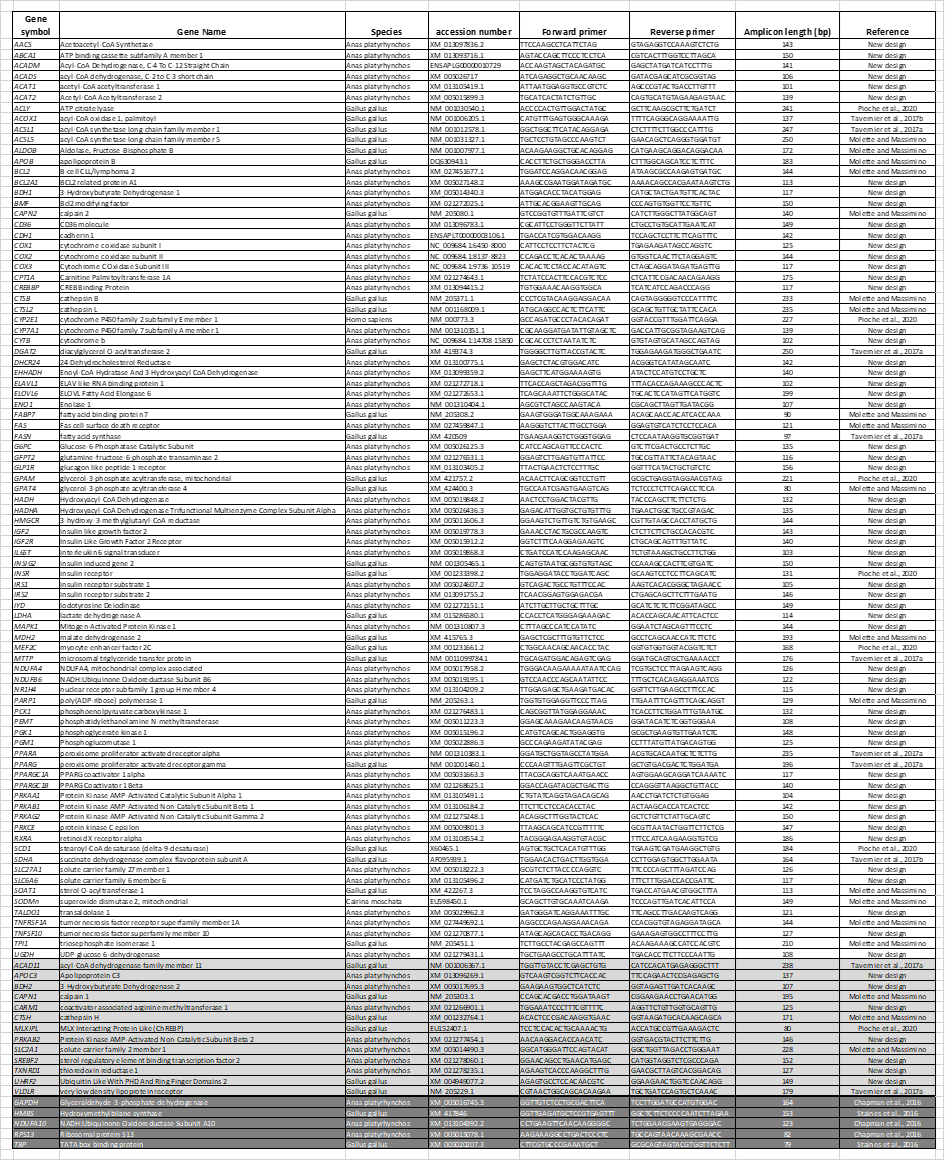


The genes targeted in the study are related to energy metabolism as well as to amino acid transport, oxidative stress, apoptotic activity and susceptibility to liver damage. Sequences were obtained either from *Anas platyrhynchos* when available, or from *Gallus gallus* on NCBI (https://www.ncbi.nlm.nih.gov/gene/) and/or Ensembl (http://www.ensembl.org/index.htm) databases. Genes showing more that 25% of missing data were removed from the study and are highlighted in light grey. The 5 potential reference genes are highlighted in dark grey. Molette and Massimino: unpublished data from Caroline Molette and William Massimino.
