# Supplementary material for: Maternal dietary methionine restriction alters the expression of energy metabolism genes in the duckling liver
Source: BMC Genomics. 2022 May 30;23:407. doi: 10.1186/s12864-022-08634-1 (PMC9150296; doi:10.1186/s12864-022-08634-1)
Supplement: Supplementary file 4 — Additional file 4: Figure 1. Correlation matrices of the transcript level of the 16 DEGs between diets and the phenotypic traits of the ducklings. [file 12864_2022_8634_MOESM4_ESM.docx]

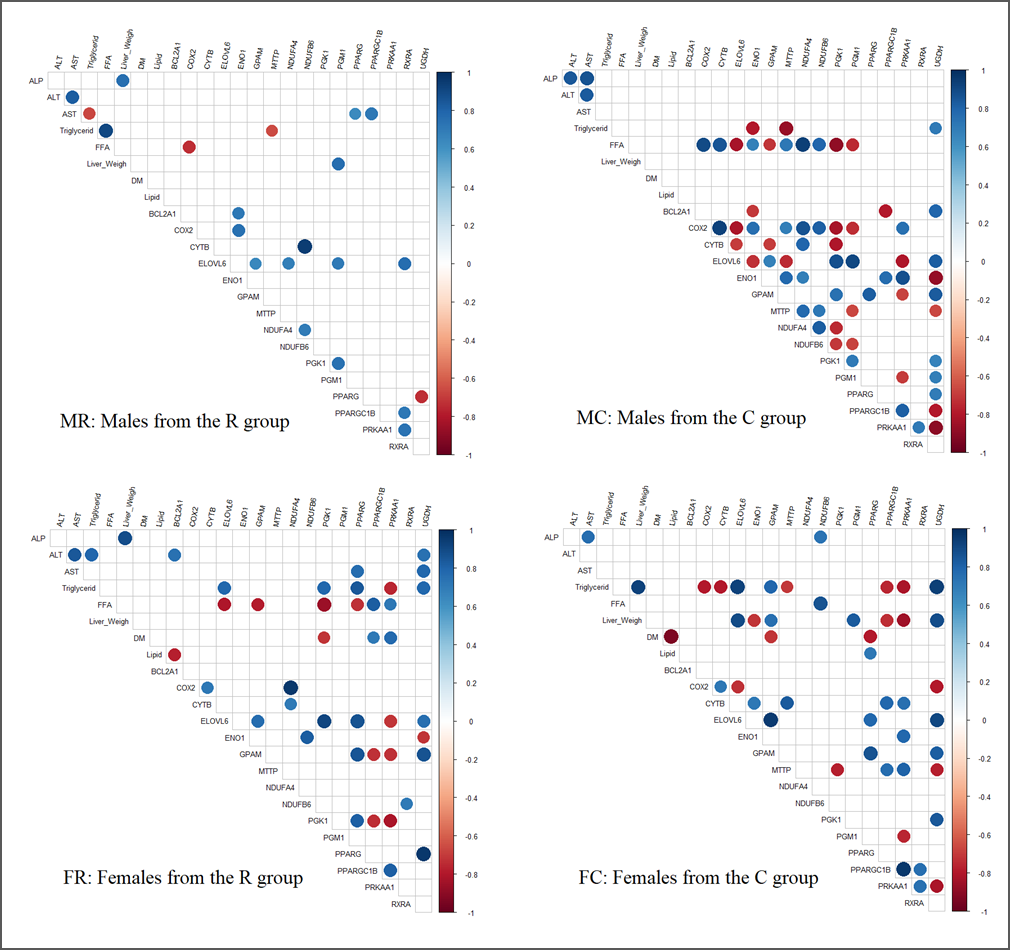


MR for males from the R group (n = 10), MC for males from the C group (n = 9), FR for females from the R group (n = 8) and FC for females from the C group (n = 8). Phenotypic traits are body weight, liver weight, percentages of liver lipids and liver dry mater (DM), plasma activities of ALP, ALT and AST, plasma cholesterol, glucose, triglyceride and free fatty acid (FFA) concentrations. The values used for the 16 DEGs were the imputed normalized expression and the values for the phenotypic data were the raw values. The color scale indicates the strength of the correlation; blue for a positive correlation and red for a negative one. Only the significant correlations (with a P-value < 0.05) were plotted.
